# Zinc Nanobiohybrid-Catalyzed Hydrolysis of *N*‑Glycosidic Bond of Uridine to Uracil and Derivatives by a Photo-Fenton Oxidation

**DOI:** 10.1021/acsomega.6c05041

**Published:** 2026-07-07

**Authors:** Carla Garcia-Sanz, Noelia Losada-Garcia, Jose M. Palomo

**Affiliations:** Instituto de Catálisis y Petroleoquímica (ICP), 16379 CSIC, c/Marie Curie 2, 28049 Madrid, Spain

## Abstract

In recent years, nanotechnology and nanobiohybrid systems have emerged as powerful platforms for applications in biomedicine, biotechnology, and catalytic chemical synthesis. In this study, a novel photocatalytic strategy was developed for the selective hydrolysis of uridine and uridine-derived substrates into uracil using a zinc-based nanobiohybrid catalyst. Different metal–enzyme nanobiohybrids were evaluated as catalysts for the hydrolysis reaction in aqueous media under mild conditions. Among the materials investigated, Zn-BIC, a nanobiohybrid containing highly crystalline Zn nanoparticles of approximately 5 nm that generate nanochannel-like structures, exhibited the highest catalytic performance and selectivity toward uridine hydrolysis. Using 10 mg of catalyst in the presence of 3% v/v H_2_O_2_ at room temperature, Zn-BIC achieved 42% uridine conversion after 1 h, corresponding to 0.42 mmol·h^–1^. The substrate selectivity of the catalyst was further assessed in the presence of other nucleosides, including deoxyadenosine and thymidine, for which negligible activity was observed, demonstrating the remarkable specificity of the system toward uridine-based substrates. Furthermore, uridine derivatives such as N4-acetylcytidine and uridine monophosphate (UMP) showed outstanding catalytic conversions of 100% (0.68 mmol·h^–1^) and 68% (6 mmol·h^–1^), respectively, under the same reaction conditions. Finally, mechanistic studies confirmed the involvement of a photo-Fenton-like pathway in the catalytic process mediated by Zn-BIC.

## Graphical abstract

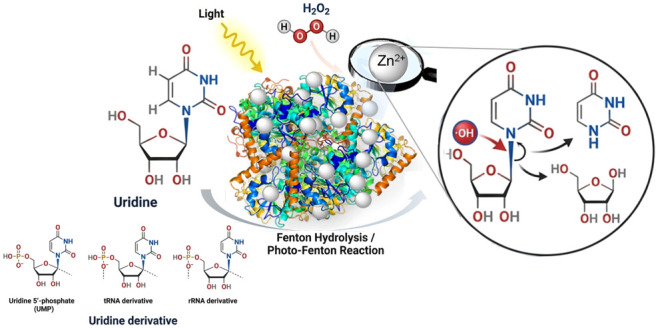

## Introduction

In recent years, nanoscience and nanotechnology have driven significant advances in the development of sustainable catalytic systems.^1^ In this context, the synthesis of transition metal nanoparticles using environmentally benign methodologies has attracted considerable attention.^1,2^ In particular, the design of enzyme–metal nanobiohybrids formed in aqueous media under mild conditions represents an efficient and green alternative to conventional approaches.^3–9^ These systems avoid the use of harsh reagents and energy-intensive processes, fully aligning with the principles of green chemistry.^7,8^

In these nanobiohybrids, the enzyme acts as both a scaffold and a stabilizing agent, promoting the in situ formation of metal nanoparticles while preventing aggregation. This strategy enables precise control over nanoparticle size and distribution, which are key factors governing catalytic performance. Moreover, the resulting materials operate efficiently in aqueous environments under mild conditions, making them particularly suitable for applications involving sensitive biomolecules.^6–8^

A particularly relevant application of these systems is the selective hydrolysis of uridine or uridine-containing residues to uracil, a transformation that mimics enzymatic processes such as those mediated by uracil-DNA glycosylase.^10,11^ This approach provides a sustainable pathway for the purification of nucleic acid samples by selectively eliminating RNA contaminants or DNA strands containing uracil, which may originate from chemical damage or enzymatic processes. Such selectivity reduces the need for multistep purification protocols and minimizes chemical waste generation, contributing to more efficient workflows in molecular biology.

The implementation of nanobiohybrid-based purification strategies is particularly relevant in forensic science, where DNA samples are often degraded, contaminated, or available only in trace amounts. The selective removal of interfering nucleic acids can significantly improve the quality and reliability of genetic analyses, enhancing downstream processes such as amplification and sequencing. From a sustainability perspective, these advances reduce sample processing steps, reagent consumption, and analytical errors, thereby increasing robustness and reproducibility in forensic workflows. Ultimately, the integration of catalytic nanotechnologies into DNA purification protocols contributes to more reliable forensic evidence while adhering to the principles of sustainable chemical design. Other applications include selective removal of amplicons (nucleic acid sequences used in amplification processes) or detection of RNA viruses in biological samples.^12–15^

Therefore, the main objective of this work is the selective hydrolysis of uridine and uridine-derived substrates to uracil using a new class of metal-based nanobiohybrids (Figure 1). The generated uracil can subsequently be detected through a selective fluorimetric method. For example, Kai et al.^12^ reported that, under optimized reaction conditions (alkaline medium, benzamidoxime, K_3_[Fe­(CN)_6_] as oxidizing agent, and heating at 90 °C for 10 min), a strong fluorescence signal is produced selectively from uracil, while other nucleosides and nucleobases present in RNA do not interfere. In addition, this method enables uracil detection at picomolar concentrations, highlighting its potential for the detection of RNA-derived uracil or genetic mutations (U incorporation in DNA).^12,16^

**Figure 1:**
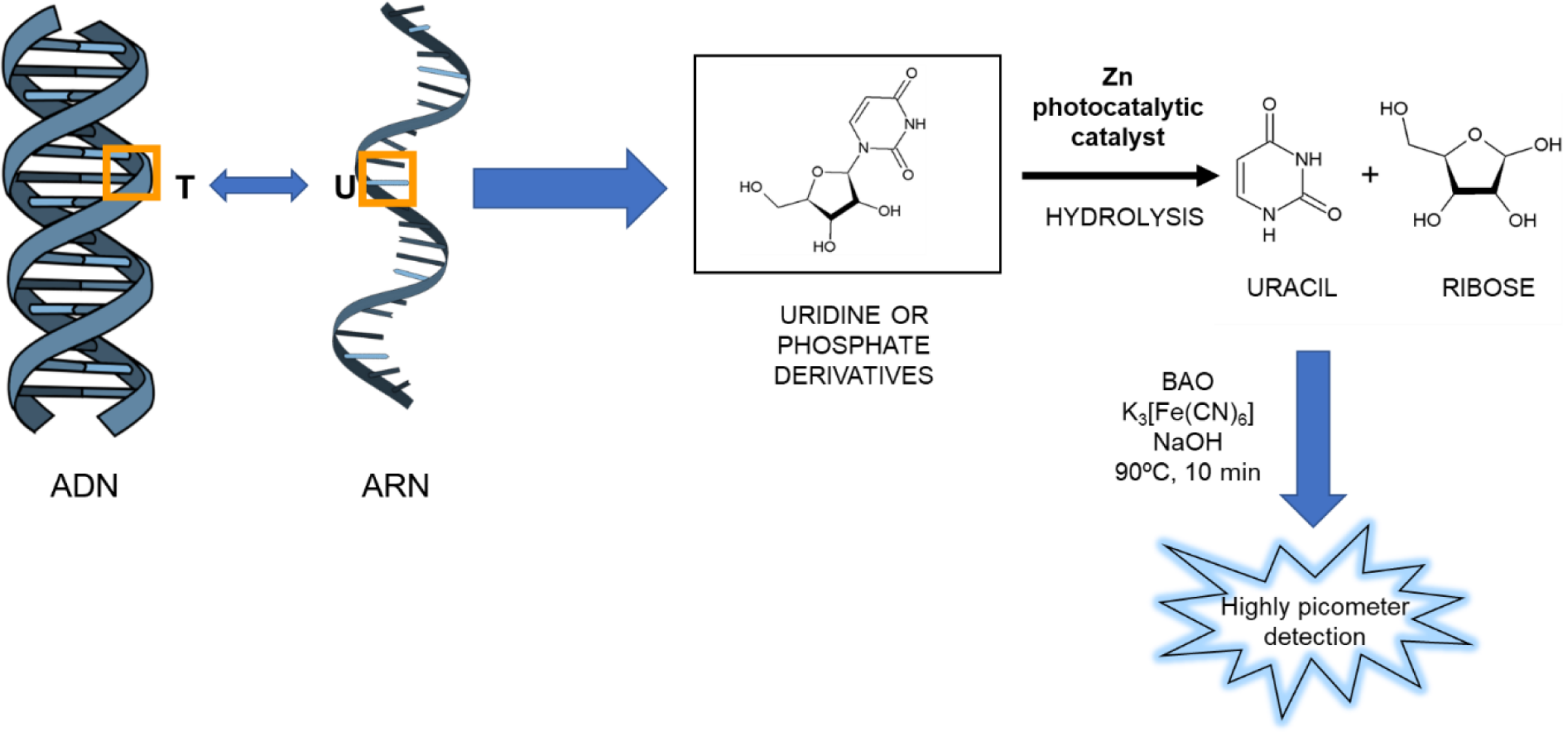
Proposed catalytic approach for the selective transformation of uridine and uridine derivatives into uracil mediated by Zn nanobiohybrid.

## Results and Discussion

### Evaluation of the Activity, Specificity, and Selectivity of Nanobiohybrids in the Uridine Hydrolysis Reaction

First, different metallic nanobiohybrids (Pd, Au, Cu, Zn) were synthesized following previously described procedures (Figure 2).^3,6,17,18^ In all cases, the catalysts were characterized, confirming the formation of distinct nanostructures within the enzyme–metal hybrids (Figure 2). The enzyme used as scaffold was lipase B from *Candida antarctica* (CALB), a well-known commercial enzyme provided by Novonesis (formerly Novozymes). This enzyme exhibits a highly stable and rigid structure over a wide pH range, making it suitable for different synthetic protocols.

**Figure 2:**
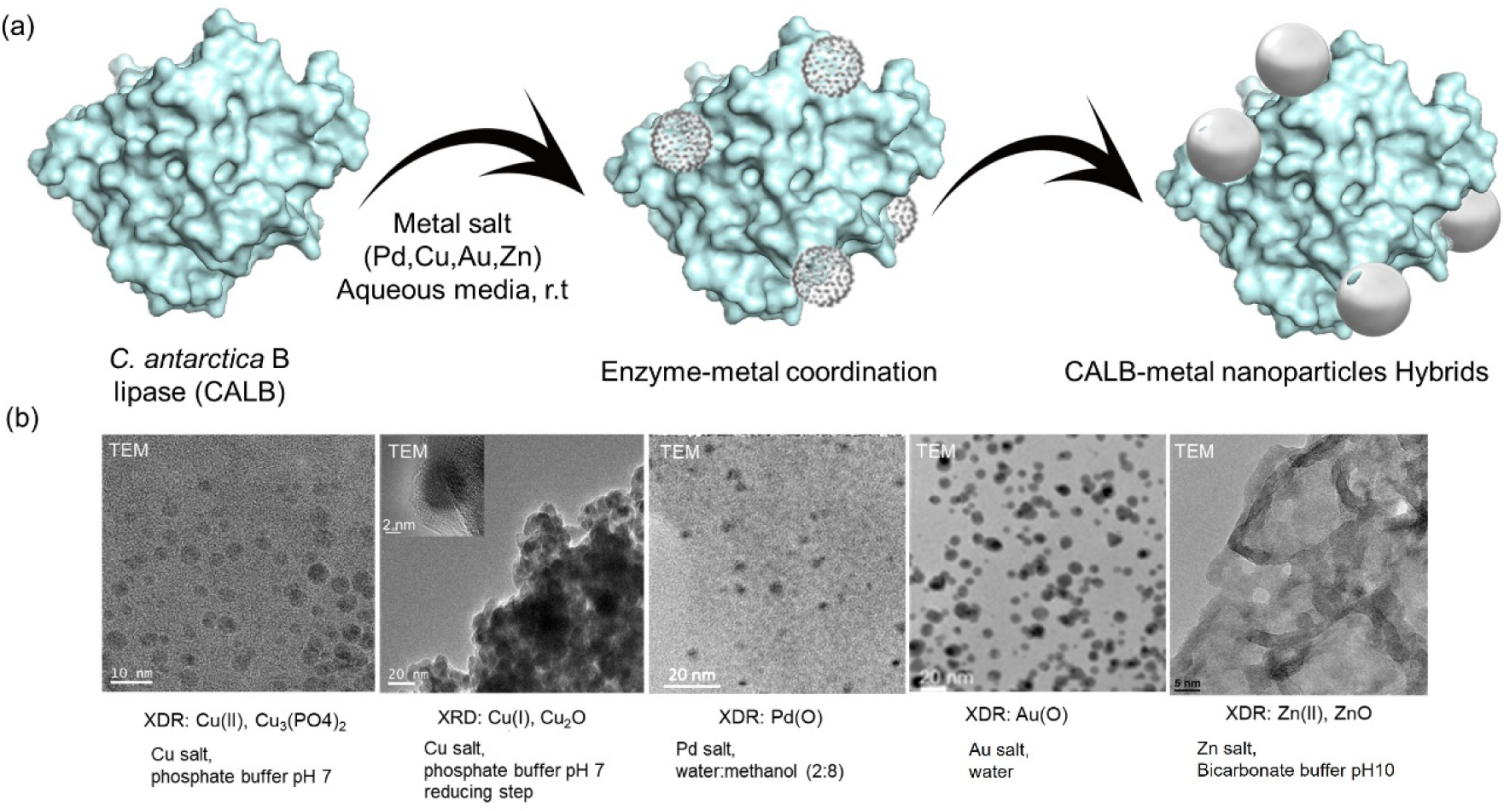
(a) Synthetic concept and design of the enzyme-metal nanoparticles hybrids strategy using different metal with CALB as scaffold. The protein structure was obtained from the Protein Data Bank (PDB code: 1TCA (CALB)) and the structure was created using PyMOL. (b) TEM and XRD characterization, experimental conditions of the different metal nanobiohybrids synthesized.

In this context, two different copper-based hybrids were prepared: one directly using copper sulfate in phosphate buffer, and a second one including a reduction step using NaBH_4_. Characterization confirmed the formation of Cu­(II) species in the first case and Cu­(I) species as Cu_2_O in the reduced system (Figure 2b). The first approach led to small metal nanoparticles embedded within a nanoflower-like mesoscale structure,^6^ whereas the reduced system produced larger nanoparticles (7–8 nm).

In the case of noble metals, the redox properties of the system allow the enzyme to act as a reducing agent, leading to the formation of Pd(0) and Au(0) nanoparticles, respectively, under mild aqueous conditions.^9,17^ In the case of palladium, palladium acetate was used in the presence of 20% (v/v) methanol, whereas gold chloride was reduced in pure aqueous medium (Figure 2b). The amount of metal salt used was also found to be important: while Cu and Au systems required 10 mg mL^–1^, Pd required only 1 mg mL^–1^.

Finally, the synthesis of the Zn-based hybrid (Zn-BIC) using zinc sulfate follows a similar base-metal protocol, employing 10 mg mL^–1^ of Zn salt. However, optimal nanomaterial formation was achieved in the presence of bicarbonate at pH 10, under alkaline conditions where CALB remains stable. This procedure resulted in the formation of ZnO nanoparticles with an average diameter of approximately 5 nm embedded within a nanochannel-like structure (Figure 2b). In all cases, the resulting hybrid systems were reproducible and stable.

The catalytic performance of the synthesized nanobiohybrids was evaluated in the hydrolytic cleavage of uridine in an aqueous medium at room temperature. The corresponding conversion values, kinetic parameters, and catalytic efficiencies determined after 24 h of reaction are summarized in Table 1. To quantify the reaction kinetics, the apparent rate constants (*k*_obs)_ were determined by fitting the experimental data to a pseudo-first-order kinetic model with respect to uridine. This model is justified by the exponential decay of the substrate over time and the large excess of catalytically active sites relative to the initial substrate concentration ([Active Sites] ≫ [Substrate]). Under these experimental conditions, the initial catalytic activity followed the trend Zn > Pd ≈ Cu_2_O > Cu_3_(PO_4_)_2_ ≫ Au, which is in strict agreement with the overall kinetic behavior observed throughout the reaction course.

**Table 1:**
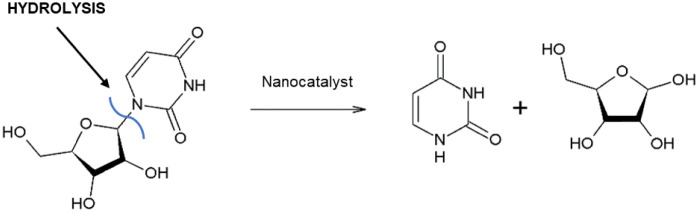
Selectivity of the Different Nanocatalysts for Uridine Hydrolysis reaction^a^

Entry	Nanocatalyst	*k*_obs_ (h^–1^)	r_avg_ (μmol h^–1^)	U (μmol h^–1^ mg^–1^ metal)	Conversion (%)	Uridine hydrolisis (%)
1	Cu_2_O NPs CALB^[6]^	0.043	0.542	0.080	65	0
2	Cu_3_ (PO_4_)_2_ NPs CALB^[6]^	0.020	0.317	0.096	38	0
3	Au(0) NPs-CALB^[18]^	0.003	0.058	0.037	7	0
4	Pd(0) NPs-CALB^[9]^	0.045	0.55	0.014	66	0
5	ZnO NPs CALB (Zn-BIC)	0.079	0.71	0.016	85	85

a Uridine 1 mM, nanocatalyst (10 mg), 20 mL solution (H_2_O), room temperature, 24 h, NPs: nanoparticles.

A consistent trend was also observed for the average reaction rate (r_avg_), which serves as a direct indicator of overall catalytic productivity. The zinc-based nanobiohybrid (Zn-BIC) exhibited the highest productivity (0.708 μmol·h^–1^), followed closely by its Pd (0.550 μmol·h^–1^) and Cu_2_O (0.542 μmol·h^–1^) counterparts, whereas the Cu_3_(PO4)_2_ and Au catalysts displayed significantly lower productivities, yielding values of 0.317 and 0.058 μmol·h^–1^, respectively. To establish a rigorous comparison regarding the intrinsic efficiency of the active sites, the catalytic activity was normalized against the metal content of each hybrid. As shown in Table 1, Zn-BIC outperformed the other materials, demonstrating a specific activity of 0.165 μmol·h^–1^·mgmetal^–1^. This represents an approximate 1.7-fold increase relative to Cu_3_(PO4)_2_ (0.096 μmol·h^–1^·mgmetal^–1^) and more than a 4-fold increase compared to Au (0.0365 μmol·h^–1^·mgmetal^–1^), highlighting the superior efficiency of the Zn sites on a mass basis.

This enhanced performance is further reflected in the total substrate conversion, where Zn-BIC achieved 85% uridine conversion within 24 h. Remarkably, extending the reaction time to 30 h allowed Zn-BIC to reach a near-quantitative conversion of over 99% toward selective uridine hydrolysis, a level of reactivity that was not matched by any of the other evaluated catalysts under identical conditions. Kinetic analysis reinforces these findings, as Zn-BIC exhibited markedly accelerated apparent kinetics compared to the Pd, Cu and Au, establishing a clear performance threshold between the zinc-based system and the less active catalysts. Taken together, these results demonstrate that Zn-BIC combines the highest reaction rate, the fastest kinetic profile, and the greatest overall catalytic productivity across the evaluated series.

A comparative analysis revealed distinct outcomes that might reflect alternative reaction pathways depending on the metallic center integrated into the nanobiohybrid. The Pd- and Cu-based catalysts may promote alternative reaction pathways that deviate from the targeted *N*-glycosidic bond hydrolysis (Table 1, entries 1, 2, and 4). These systems might actively mediate the oxidation of the pyrimidine ring, which could lead to the formation of multiple oxidative side products, suggesting that oxidative transformations may compete effectively with hydrolytic cleavage under the utilized reaction conditions.^19^ In contrast, the Au-based hybrid exhibited negligible reactivity, showing no meaningful contribution to either hydrolytic or oxidative pathways (Table 1, entry 3).

Conversely, the Zn-BIC nanobiohybrid displayed highly chemoselective behavior. The conversion of uridine over Zn-BIC appeared to proceed predominantly via *N*-glycosidic bond cleavage, selectively yielding the corresponding nucleobase. Notably, high-performance liquid chromatography (HPLC) analysis did not detect any significant accumulation of oxidative byproducts above the analytical limits of detection (LOD). In summary, Zn-BIC exhibits a strong preference for nucleobase formation via selective hydrolysis, potentially distinguishing itself as a highly efficient and chemoselective catalyst within the evaluated nanobiohybrid series.

Subsequently, the selectivity of the Zn-BIC nanobiohybrid was evaluated in the hydrolytic degradation of uridine in the presence of other nucleosides, namely deoxyadenosine and thymidine. In this context, selectivity was assessed by comparing the extent of degradation of each nucleoside under identical reaction conditions. Figure 3 shows the percentage of degradation of the different nucleosides after 24 h of reaction. As observed, negligible degradation of thymidine and deoxyadenosine was detected compared to uridine, which reached 85% conversion under the same conditions

**Figure 3:**
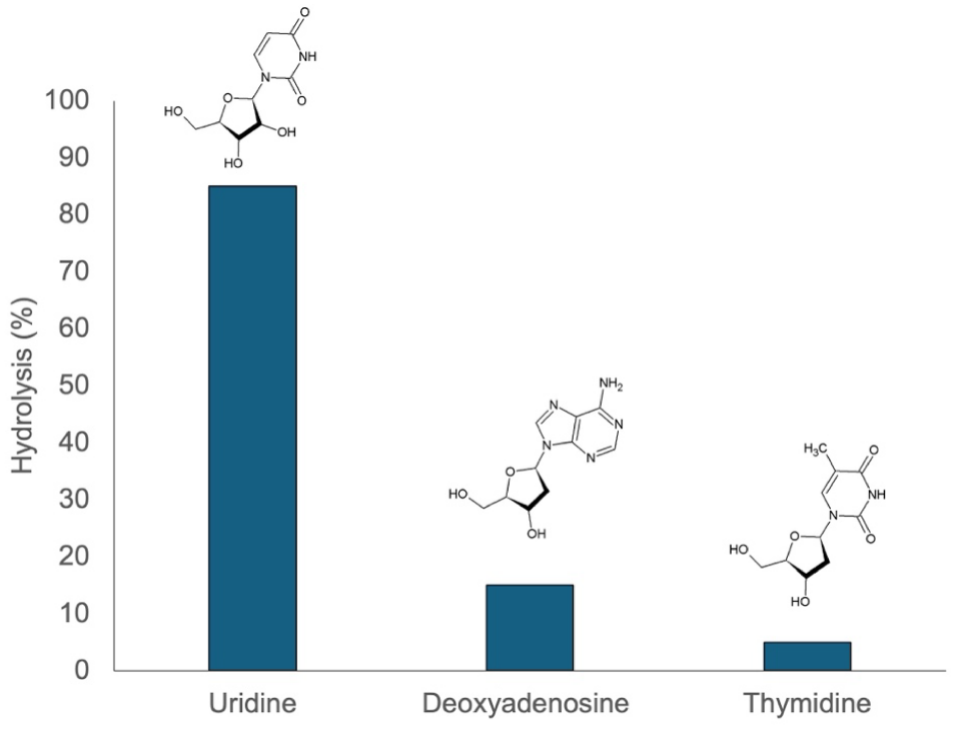
Selective degradation of nucleosides by Zn-BIC after 24 h and r.t.

This behavior can be rationalized based on the structural differences between the nucleosides. In thymidine and deoxyadenosine, the nitrogenous base is attached to a 2′-deoxyribose moiety, whereas uridine contains a ribose unit bearing a 2′-hydroxyl group. The presence of these 2′-OH groups may influence the electronic distribution and hydrogen-bonding environment of the glycosidic bond, facilitating its cleavage and resulting in higher susceptibility to degradation under the studied conditions.

Therefore, the Zn-BIC nanocatalyst shows preferential reactivity toward uridine compared to other nucleosides, indicating substrate-dependent selectivity. In addition, the transformation proceeds predominantly through hydrolytic glycosidic bond cleavage, without any evidence of oxidation of the pyrimidine ring under the studied conditions.

### Optimization of the Hydrolysis Reaction

In this section, we optimized the experimental conditions of the reaction. First, the effect of hydrogen peroxide (H_2_O_2_) addition on the hydrolysis reaction was studied. Hydrogen peroxide is widely recognized as a powerful green oxidant, and its selection in this study is based on previous work by our group using iron and copper nanocatalysts, where it was shown to accelerate selective oxidation reactions for the degradation of target compounds.^5,6,20,21^ Therefore, to achieve the highest degree of uridine hydrolysis and, consequently, uracil formation in the shortest possible time, the catalytic performance of Zn-Bic in the presence of hydrogen peroxide was evaluated.

For this purpose, the reaction was carried out using different concentrations of H_2_O_2_ (0, 1, 2, 3, and 4% v/v) at room temperature, and the effect on conversion yield was assessed. As shown in Figure 4a, hydrolytic conversion was around 5% after 1 h of incubation in the absence of hydrogen peroxide. However, hydrolysis yields of 10% and 18% were obtained upon addition of 1% and 2% (v/v) H_2_O_2_, respectively (Figure 4a). The addition of up to 3% (v/v) H_2_O_2_ further increase the efficiency of the Zn nanocatalyst, leading to up to 42% selective conversion of uridine into uracil and ribose after 1 h, representing more than a 5-fold increase compared to the reaction performed without oxidant.

**Figure 4:**
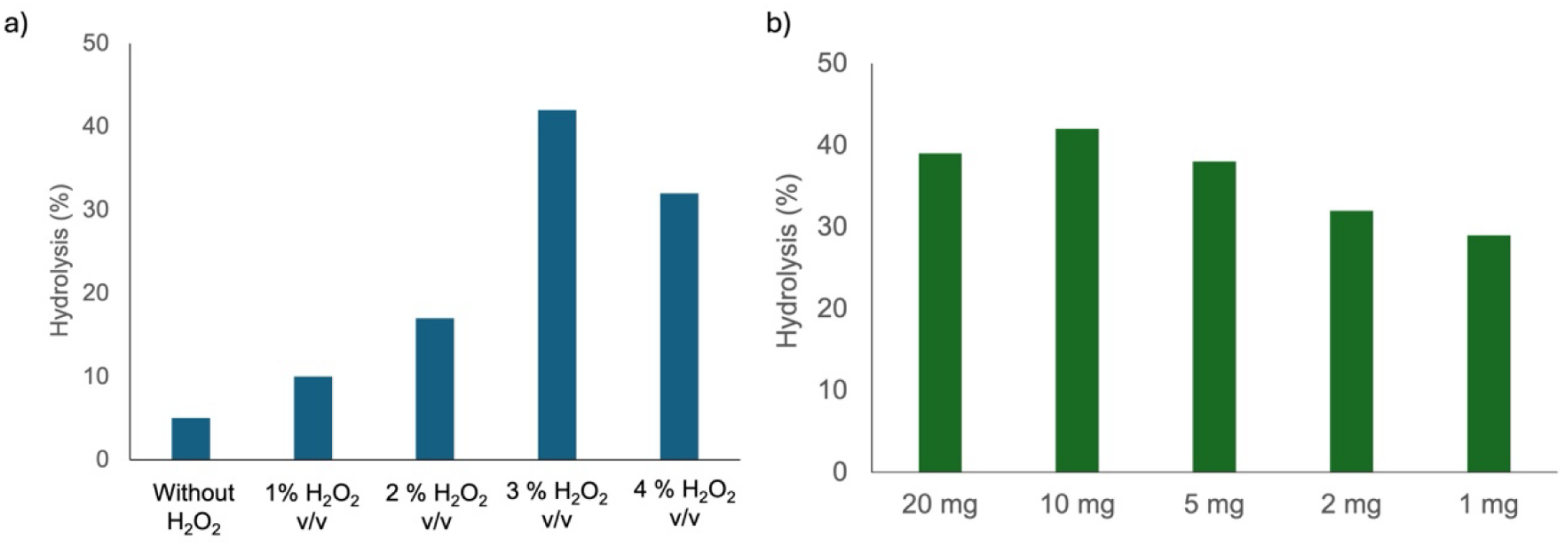
a) Effect of H_2_O_2_ in the uridine (10^–3^ mol/L) hydrolysis. b) Percentage of uridine (10^–3^ mol/L) hydrolysis with different amounts of Zn-BIC for 1 h reaction time.

An excess of hydrogen peroxide did not further enhance catalytic performance; with 4% H_2_O_2_, hydrolysis decreased to 32%. Thus, 3% (v/v) H_2_O_2_ was selected as the optimal additive for subsequent studies.

In the next step, the effect of varying the amount of Zn-Bic (1–20 mg of catalyst) on the hydrolysis of uridine in aqueous solution was investigated at 1 h reaction time (Figure 4 b). The highest conversion (42%) was obtained using 10 mg of nanocatalyst. However, a similar value was observed with 5 mg (39%), indicating only a marginal improvement at higher catalyst loading. In contrast, lower catalyst amounts led to decreased hydrolysis yields specifically, using 1 mg of nanocatalyst resulted in less than 30% conversion These results indicate that the relationship between catalyst loading and hydrolysis yield is not linear, and that increasing the amount of Zn-Bic beyond a certain threshold does not further improve conversion.

Based on these results, the optimal conditions for the hydrolysis reaction were determined to be 10 mg of Zn-Bic nanocatalyst and 3% (v/v) H_2_O_2_.

### Evaluation of the Hydrolysis of Uridine and Related Nucleoside Derivatives

In order to mimic conditions similar to those present in RNA sequences, the degradation of uridine and related RNA nucleosides catalyzed by Zn-BIC was evaluated using 5′-uridine monophosphate (UMP), N4-acetylcytidine (ac^4^ C), and cytidine. UMP is a phosphate ester of uridine and a canonical RNA nucleotide.^22^ N4-acetylcytidine is a naturally occurring post-transcriptional modification found in tRNA and rRNA, where it plays an important role in translational fidelity and RNA stability.^23–25^ Cytidine is a canonical RNA nucleoside structurally related to uridine, differing only in the exocyclic amino group at the C4 position of the pyrimidine ring.^26^ Structurally, these nucleosides introduce electronic perturbations relative to uridine, either through phosphorylation, acetylation, or base modification, which can influence the electronic distribution, hydrogen-bonding pattern, and solvation environment of the nucleoside framework.

The influence of these structural variations was evaluated by comparing their degradation behavior under identical conditions (Figure 5). N4-acetylcytidine exhibited complete conversion (100%), whereas UMP reached 68%, both exceeding that of uridine (42%). Cytidine showed partial conversion (29%) under the same conditions. This reactivity trend can be rationalized in terms of electronic effects, where the electron-donating amino group in cytidine increases the electron density of the pyrimidine ring, thereby reducing its susceptibility toward oxidative activation under these conditions. This suggests that modifications at either the nucleobase or the sugar–phosphate backbone can significantly modulate *N*-glycosidic bond reactivity.^27–29^ In particular, electronic and hydrogen-bonding effects may influence charge distribution at the glycosidic linkage and alter the stability of the corresponding transition state for bond cleavage. Additionally, in UMP, the phosphate group introduces electrostatic and solvation effects that may further impact substrate–catalyst interactions and accessibility of the reactive site.^30,31^

**Figure 5:**
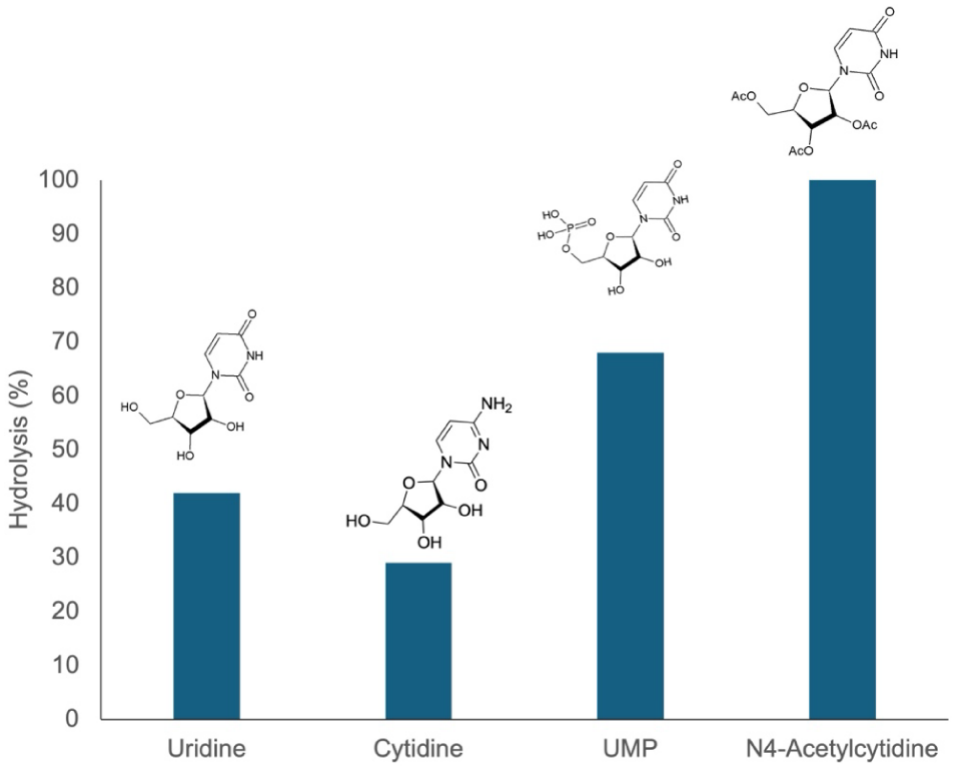
Hydrolysis percentage of uridine, cytidine, N4-acetylcytidine, and UMP 10^–3^ mol/L in 1 h reaction time, 10 mg Zn-BIC, 3% H_2_O_2_ (v/v), r.t.

The higher reactivity observed for N4-acetylcytidine relative to UMP is attributed to the stronger electron-withdrawing nature of the acetyl substituent, which more effectively modulates the electronic properties of the nucleobase and enhances glycosidic bond lability compared to phosphorylation alone.^32^ These observations are consistent with literature reports highlighting the sensitivity of nucleoside reactivity to electronic perturbations that govern bond stability and hydrolysis kinetics.^33^ Among the derivatives studied, UMP was selected for further hydrolysis studies due to its phosphate group, which renders it more representative of native RNA structural features.

Subsequently, the hydrolysis reaction was evaluated using UMP. As shown in Figure 6 a, at a concentration of 10^–3^ mol L^–1^, the conversion reached 48% after 10 min and increased to 68% after 1 h. The 10 min time point was selected as a reference, as approximately half of the substrate is converted to uracil within this period, providing a suitable conversion level for analytical detection, for example by fluorometric methods.^12^

**Figure 6:**
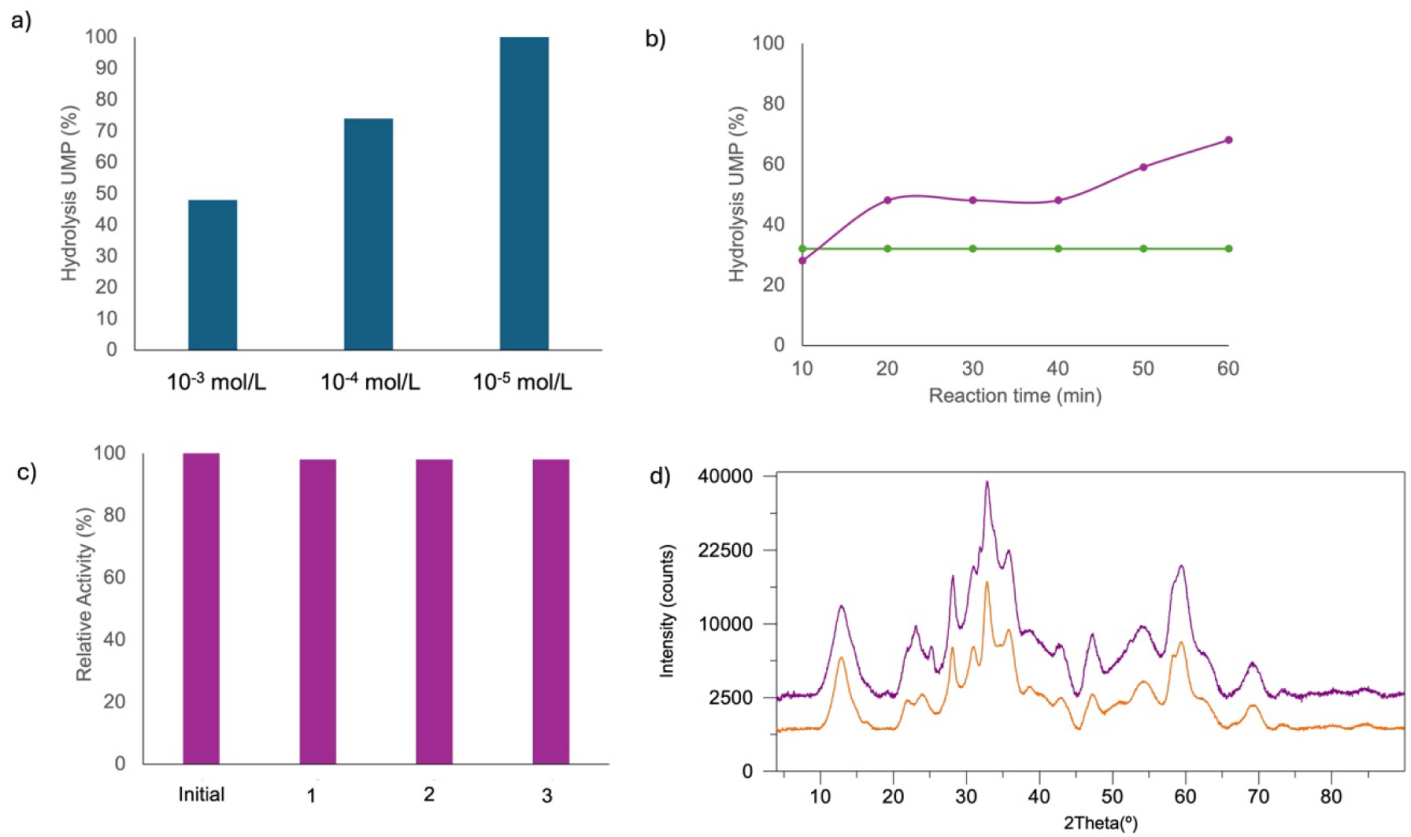
a) Hydrolysis percentage obtained at different UMP concentrations after 10 min of reaction at 25 °C; b) Hydrolysis of UMP (10^–3^ mol L^–1^) catalyzed by Zn-BIC at 25 and 50 °C; c) Reusability study of the Zn-BIC catalyst over three consecutive catalytic cycles, showing the relative activity with respect to the initial cycle; d) XRD patterns of the Zn-BIC catalyst before reaction (purple line) and after the recycling experiments (orange line).

The effect of substrate concentration was then investigated by decreasing the initial UMP concentration to 10^–4^ and 10^–5^ mol L^–1^. As shown in Figure 6 b, the extent of hydrolysis increased with decreasing concentration. At 10^–4^ mol L^–1^, approximately 75% conversion was observed, whereas at 10^–5^ mol L^–1^ complete conversion was achieved within 10 min. These results suggest that at sufficiently low substrate concentrations, the system approaches quantitative hydrolysis within short reaction times.

Next, the effect of temperature was assessed to evaluate its influence on UMP conversion. Reactions were performed at 50 °C and compared with those at room temperature (25 °C). Prior to the catalytic experiments, UMP stability in the presence of H_2_O_2_ (3% v/v) at 50 °C was confirmed by HPLC analysis (data not shown). Under catalytic conditions, a constant conversion of 32% was observed at 50 °C, independent of reaction time. Notably, this value was lower than that obtained at 25 °C (48%), indicating that increasing temperature does not enhance the reaction and is therefore detrimental to catalytic performance under the studied conditions.

Finally, the recyclability of the Zn-BIC catalyst was evaluated through consecutive uridine hydrolysis reactions (Figure 6c). The catalytic performance after each cycle was compared with that obtained in the first reaction after 30 min, which was taken as the reference value (100% relative activity). The catalyst retained 98% of its initial activity after three catalytic cycles. This slight decrease is mainly attributed to catalyst loss during the recovery and washing steps between cycles rather than to intrinsic catalyst deactivation, confirming its recyclability. Furthermore, XRD analysis of the recovered catalyst after the recycling experiments revealed that the crystalline structure of Zn-BIC remained unchanged, thus confirming its structural stability throughout the catalytic process (Figure 6 d).

### Mechanism of the Zn-BIC-Catalyzed Reaction

To elucidate the possible mechanism of the degradation reaction catalyzed by the Zn-BIC nanobiohybrid, different mechanistic probes were employed. First, the potential involvement of radical species was evaluated using TEMPO (2,2,6,6-tetramethylpiperidin-1-yl)­oxyl, a well-known radical scavenger. TEMPO acts as a radical-trapping agent capable of quenching reactive radical intermediates generated during the reaction. Accordingly, if the degradation proceeds through a radical pathway, a significant decrease in conversion would be expected in the presence of TEMPO due to suppression of radical propagation processes.

Under TEMPO-containing conditions, the conversion decreased to 5% after 10 min, compared with 48% in the absence of TEMPO (Figure 7a). This strong inhibition indicates the involvement of radical species in the catalytic process and provides evidence for the involvement of radical intermediates within the overall catalytic process.

**Figure 7:**
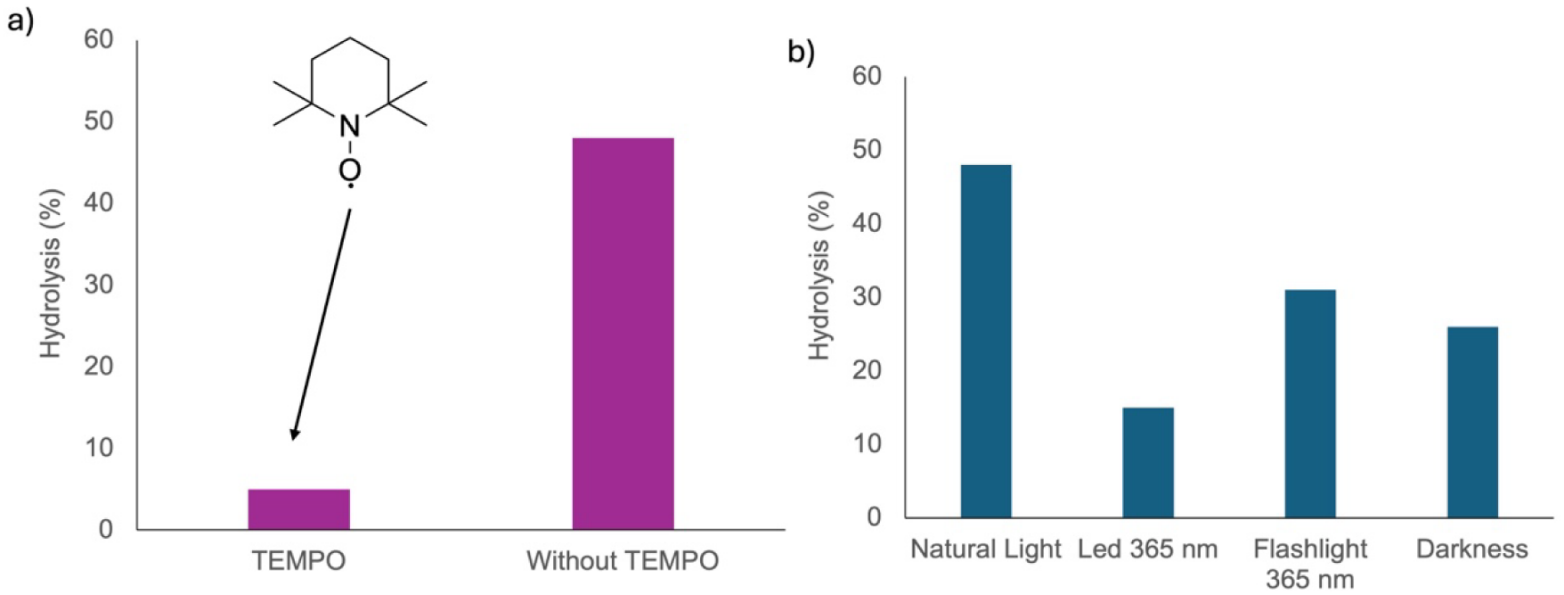
a) Reaction with and without TEMPO b) Photocatalytic reaction: natural light, flashlight UV 365 nm, lamp UV 365 nm and darkness.

In addition, the possible contribution of photocatalytic processes was investigated, considering the known photocatalytic properties of Zn-based catalysts.^34,35^ The reaction was performed for 10 min under different irradiation conditions (Figure 7b), while keeping all other experimental parameters constant. Under natural sunlight, the highest conversion (48%) was observed. Lower conversions were obtained under artificial UV irradiation, using a 5 W UV flashlight (365 nm) and a 6 W UV lamp (365 nm), which yielded 31% and 26% conversion, respectively. In contrast, in the absence of light (dark conditions), the conversion decreased significantly to 15%, confirming that light irradiation enhances the catalytic efficiency of the Zn-BIC system and evidencing a clear light-dependent behavior.

These results indicate that light irradiation enhances the catalytic activity of the Zn-BIC system and suggest the involvement of a photoassisted oxidative pathway. In the presence of hydrogen peroxide, these processes might be consistent with a photo-Fenton-like mechanism involving the generation of reactive oxygen species (ROS), particularly hydroxyl radicals (•OH), under catalytic and irradiated conditions.^36,37^

A plausible mechanistic proposal is depicted in Figure 8. Under light irradiation, electron–hole pairs are generated within the semiconductor component of the hybrid material. Photoexcited electrons (e^–^) and photogenerated holes (h^+^) can participate in redox reactions at the catalyst surface, promoting the activation of hydrogen peroxide and leading to the formation of reactive oxygen species such as hydroxyl radicals (•OH).^38,39^ These highly reactive species are responsible for the oxidative cleavage of the nucleoside framework.

**Figure 8:**
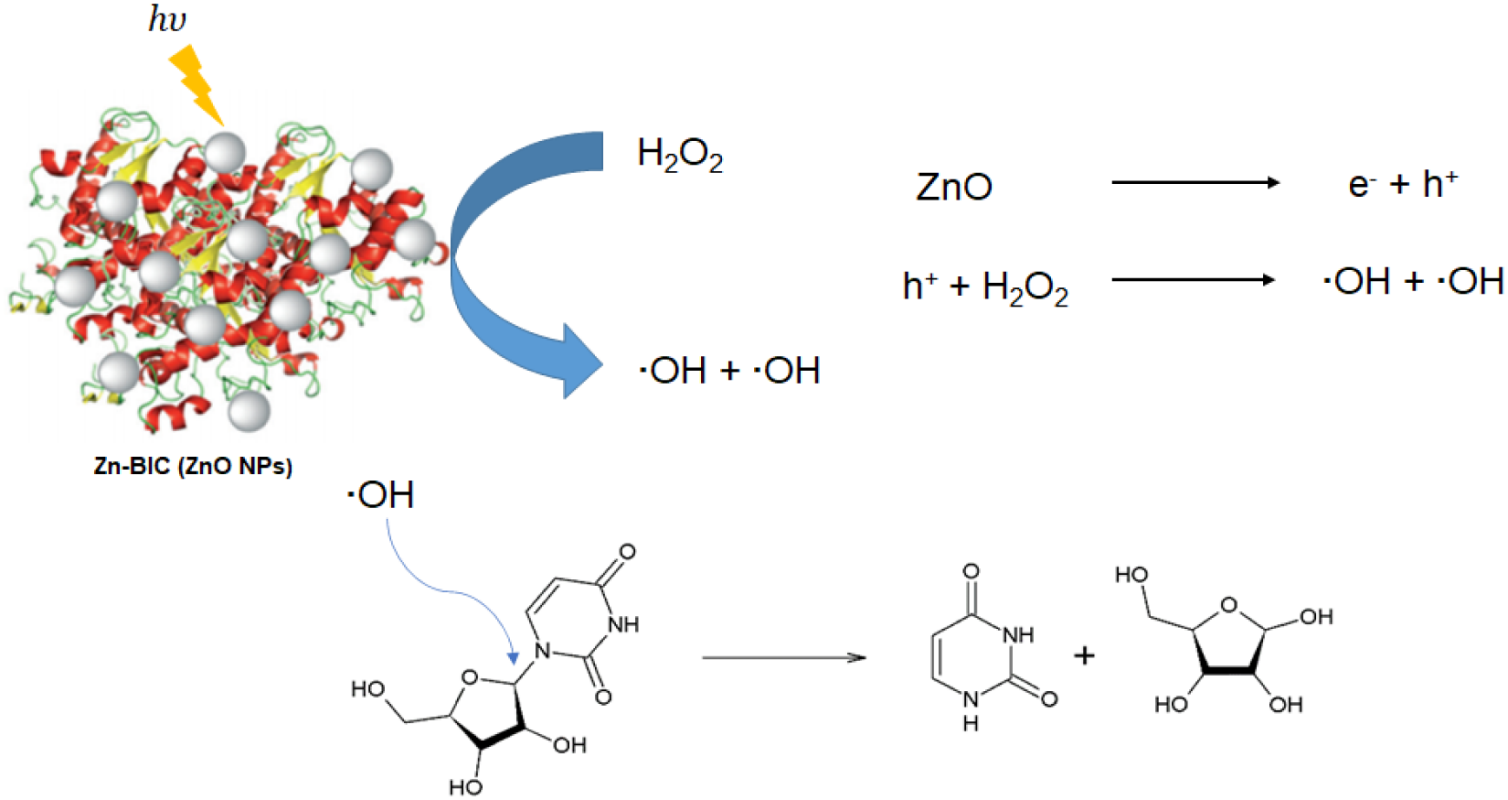
Proposed reaction mechanism for the reaction.

In this context, the cleavage of uridine proceeds predominantly through oxidative radical-mediated pathways rather than a pure hydrolytic mechanism, leading to the rupture of the *N*-glycosidic bond and the subsequent formation of the corresponding nucleobase (uracil) and sugar-derived fragments.

## Conclusions

An efficient and highly chemoselective catalytic method has been successfully developed for the hydrolysis of uridine and its derivatives using a zinc oxide-based nanobiohybrid (Zn-BIC). Operating under mild conditions at room temperature in the presence of H_2_O_2_ (3% v/v), the system achieved a 42% conversion of uridine (0.42 mmol·h^–1^) within 1 h, demonstrating remarkable selectivity as no significant degradation was observed for other pristine nucleosides. Furthermore, the catalytic scope of Zn-BIC was significantly enhanced when evaluating chemically modified derivatives. Specifically, N4-acetylcytidine reached 68% conversion (0.68 mmol·h^–1^). in 1 h, while the phosphate derivative, uridine 5′-monophosphate (UMP), underwent exceptionally rapid and complete transformation, reaching 100% conversion (6 mM·h^–1^) in just 10 min. Mechanistic investigations suggest that the cleavage of uridine proceeds through a photoassisted radical oxidative pathway, which is consistent with a photo-Fenton-like process driven by reactive oxygen species (ROS) generated under irradiation.

Looking forward, this catalytic system holds great promise for diverse biotechnological and biomedical applications. In molecular biology, it may enable the selective degradation of RNA contaminants in DNA samples, potentially contributing to improved nucleic acid purification strategies for analytical purposes. Additionally, the developed method could serve as a valuable tool for studying uracil-related DNA modifications and mutational processes. Beyond analysis, this platform may offer a novel conceptual approach for the detection, processing, or inactivation of RNA-containing biological systems, including RNA viruses. Finally, future studies should explore the broader catalytic scope of this material, particularly its potential activity toward glycosidic bonds in *N*-glycosylated proteins, opening new avenues in proteomics and therapeutic development.

## Experimental Section

### General

Lipase B from *Candida antarctica* (CALB) solution (Lipozyme-CALB) was purchased from Novonesis (formerly Novozymes, Copenhagen, Denmark). Hydrogen peroxide (33%) was obtained from Panreac (Barcelona, Spain). Zinc sulfate heptahydrate (ZnSO_4_·7H_2_O), uridine, thymidine, uridine monophosphate (UMP), and N4-acetylcytidine were purchased from Sigma-Aldrich (St. Louis, MO, USA). Deoxyadenosine was supplied by Carbosynth (Berkshire, United Kingdom). TEMPO was obtained from Novabiochem/Merck (Darmstadt, Germany). HPLC-grade acetonitrile was purchased from Scharlab (Barcelona, Spain).

### Synthesis and Characterization of Zn-BIC Nanobiohybrid

General scheme of the synthesis of nanobiohybrid has been followed (Figure 9). In particular, 1.8 mL of commercial CALB enzyme dilution (9 mg lipase/mL) was added to 60 mL of 0.1 M sodium bicarbonate buffer pH = 10 in a 250 mL glass vessel containing a small magnetic stirrer. Next, 600 mg of ZnSO_4_·7H_2_O (10 mg/mL) was added to the protein dilution and kept under stirring for 16 h. Then, 6 mL of a 1.2 M aqueous solution of NaBH_4_ (in two times of 3 mL) was added, obtaining a final concentration of 0.12 M sodium borohydride in the mixture. The mixture was reduced for 15 min. After incubation, it was centrifuged at 8000 rpm for 10 min. The precipitate obtained was resuspended in 10 mL of water, washed and centrifuged again at 8000 rpm for 10 min, and the supernatant was removed. This process was repeated two more times. Finally, the precipitate was resuspended in 2 mL of water, frozen in liquid N_2_ and lyophilized for 16 h. The synthesized nanobiohybrid was obtained as a white powder and named Zn-BIC.

**Figure 9:**
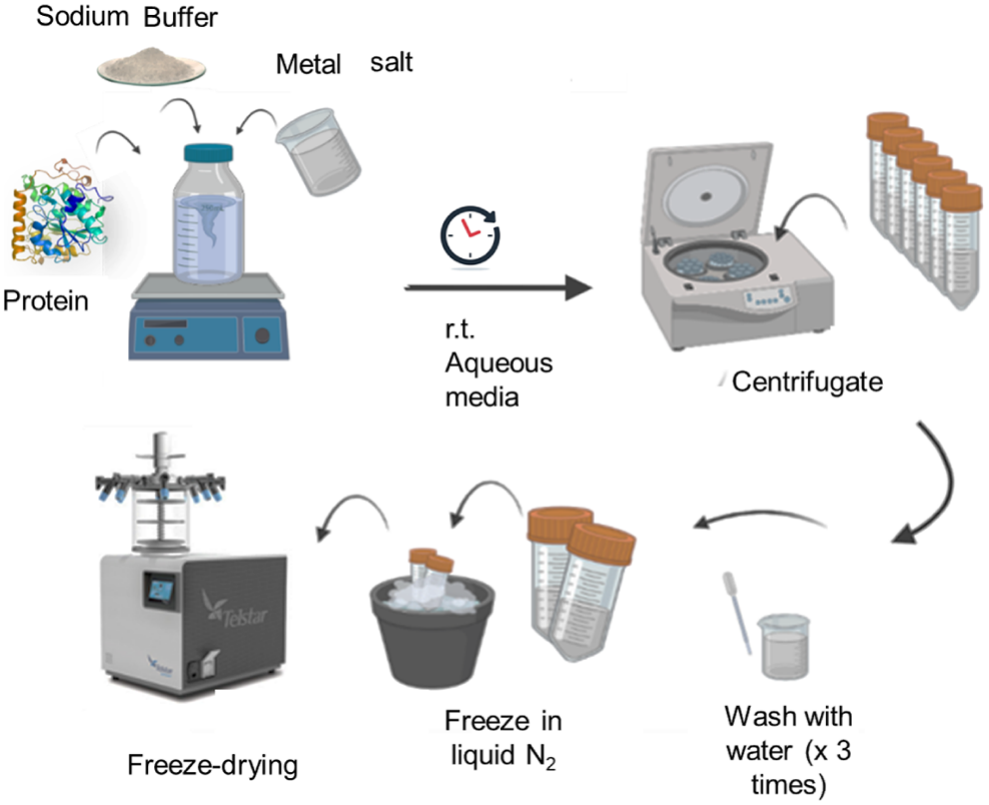
Schematic image of the synthetic procedure of enzyme-metal nanoparticles hybrids.

Characterization of the material was performed to corroborated the previously results,^14^ showing by XRD the presence of ZnO species, nanoparticles confirmed by TEM and 51% (w/w) in Zn content by ICP-OES.

### General Procedure for the Hydrolysis of Nucleosides and Derivatives

For the hydrolysis or oxidation of nucleosides, a 10^–3^ mol L^–1^ solution of uridine, deoxyadenosine, or thymidine was prepared in 20 mL of distilled water at room temperature. Subsequently, 10 mg of the corresponding nanocatalyst was added under constant stirring. After the desired reaction time, the reaction mixture was centrifuged to remove the catalyst, and the resulting supernatant was collected. The degradation of the nucleosides was determined by HPLC analysis of the supernatant.

The hydrolysis reaction of uridine and its derivatives, namely uridine 5′-monophosphate (UMP) and N4-acetylcytidine, was performed following the same procedure using substrate concentrations ranging from 10^–3^ to 10^–5^ mol L^–1^ in 20 mL of aqueous H_2_O_2_ solutions containing between 0 and 4% (v/v) hydrogen peroxide. The reaction was initiated by adding 10 mg of the Zn-BIC catalyst.

To investigate the reaction mechanism, the same reaction was carried out in the presence of 0.1 mmol of TEMPO (2,2,6,6-tetramethylpiperidin-1-yl)­oxyl, corresponding to 48 mg of TEMPO-bound polymer.

### Kinetic Analysis

Kinetic parameters were determined by monitoring the concentration of nucleosides over time by HPLC. Apparent rate constants (kobs) were obtained assuming pseudo-first-order kinetics with respect to the nucleoside, under conditions of excess catalytic sites relative to substrate concentration. The data were fitted to the integrated first-order rate equation:

$$\ln\left(\frac{C_{0}}{C_{t}}\right)=k_{obs}\times t$$

where *C*_0_ and *C_t_* are the nucleoside concentrations at time zero and time *t*, respectively.

Average reaction rates (r_avg_) were calculated from the initial linear region of product formation versus time and expressed as mM·h^–1^. All kinetic experiments were performed at room temperature, and values reported correspond to at least two independent measurements with good reproducibility.

### Analytical Determination of the Compounds

The analysis of the compounds was performed using a high-performance liquid chromatography (HPLC) system composed of a PU-4180 HPLC pump coupled to a UV-4075 UV–Vis detector (JASCO Corporation, Tokyo, Japan). Data acquisition and chromatographic analyses were carried out using ChromNAV 2.0 software (JASCO Corporation, Tokyo, Japan). All analyses were performed at 25 °C.

For thymidine and deoxyadenosine analyses, a reverse-phase C18 column (150 × 4.6 mm, 5 μm particle size; Análisis Vinílicos S.L., Tomelloso, Spain) was used. The mobile phase consisted of 80% (v/v) Milli-Q water and 20% acetonitrile (ACN) at a flow rate of 0.4 mL min^–1^. Detection wavelengths were set at 254 nm for deoxyadenosine and 225 nm for thymidine. Under these conditions, the retention times were 5.0 min for deoxyadenosine and 4.1 min for thymidine.

For uridine and N4-acetylcytidine analyses, a Kromasil C8 reverse-phase column (150 × 4.6 mm, 5 μm particle size; Eka Chemicals AB, Bohus, Sweden) was employed. The mobile phase consisted of 50% (v/v) acetonitrile in Milli-Q water at a flow rate of 0.4 mL min^–1^. UV detection was performed at 260 nm. Under these conditions, the retention times were 3.3 min for uridine, 5.1 min for N4-acetylcytidine, 3.5 min for uracil, and 3.6 min for H_2_O_2_.

For uridine 5′-monophosphate (UMP), the chromatographic conditions were modified to avoid phosphate aggregation on the column. A flow rate of 1.0 mL min^–1^ and a mobile phase consisting of 70% (v/v) Milli-Q water and 30% acetonitrile were used, with UV detection at 260 nm. Under these conditions, the retention times were 1.1 min for UMP and 1.6 min for H_2_O_2_.

In all cases, 100 μL of sample was diluted in 1 mL of the corresponding mobile phase prior to injection. Product identification was assigned by HPLC comparison with authentic standards under identical chromatographic conditions.
